# Effectiveness of clotting factor replacement therapy after antivenom treatment on coagulopathic envenomation following green pit viper bites: a retrospective observational study

**DOI:** 10.1186/s12873-022-00569-w

**Published:** 2022-01-19

**Authors:** Liangbo Zeng, Qing Liang, Zijing Liang, Jieyun Han, Miaozhu Wu, Rong Liu, Xida Wang

**Affiliations:** Department of Emergency Medicine, First Affiliated Hospital of Guangzhou Medical University, Guangzhou, 510120 China

**Keywords:** Antivenom, Clotting factor replacement, Coagulopathy, Snakebites, *Trimeresurus*

## Abstract

**Background:**

Green pit vipers (GPVs), namely *Trimeresurus albolabris* and *Trimeresurus stejnegeri* accounts for most snakebites in Southern China. Green pit viper venom contains thrombin-like enzymes, resulting in defibrination syndrome. Using of clotting factor replacement after antivenom administration is controversial. The objective of this study was to investigate the effects of clotting factor replacement in coagulopathic patients with *T. albolabris* and *T. stejnegeri* bites after antivenom administration.

**Methods:**

We retrospectively reviewed 123 patients who were bitten by *T. albolabris* and *T. stejnegeri* and were admitted to the Emergency Department of a hospital in Guangzhou, Southern China, from 2013 to 2019. Recovery of prothrombin time (PT) and fibrinogen level were compared among (1) fresh-frozen plasma (FFP) group; (2) cryoprecipitate (cryo) group; (3) FFP and cryo group; and (4) control group after antivenom administration.

**Results:**

The incidence of coagulopathy was 31%. Persistent and late coagulopathy were the most common patterns among four groups. The median reduction in PT was 20.1 ± 31.2 s for FFP and cryo group. The median increase in fibrinogen level was very small: 0.05 ± 0.20 g/L for FFP group, 0.09 ± 0.37 g/L for cryo group and 0.07 ± 0.31 g/L for FFP and cryo group, respectively. The percentage of unimproved PT was markedly higher in the FFP and cryo group than the control group (*P* = 0.01 by log-rank test, *P* = 0.02 by Gehan-Breslow-Wilcoxon test). The percentage of unimproved fibrinogen level tended to be worse in the FFP and cryo group than the control group, but the different was marginal (*P* = 0.05 by Gehan-Breslow-Wilcoxon test, *P* = 0.07 by log-rank test). A total of 7.8% (7/90) of the patients in the clotting factor replacement groups developed anaphylaxis and heart failure.

**Conclusion:**

There is no improvement in coagulopathy profile in patients with *T. albolabris* and *T. stejnegeri* bites who received clotting factor replacement after antivenom administration. But the results from GPVs may not be generalized to other species of venomous snakes.

## Background

There are more than 30 species of green pit viper (GPV) widely distributed in Asia [1–3]. Six of the GPV species are found in China, but only *T. albolabris* and *T. stejnegeri* are commonly found and of medical importance in Guangdong Province, Southern China [4]. Of note, *T. albolabris* also accounts for most snakebite victims in Hong Kong [5], while *T. stejnegeri* bites are more common in Taiwan [6].

Green pit viper venoms contain thrombin-like enzymes that consume fibrinogen, which results in severe defibrination syndrome [7, 8]. Venom kinetics revealed that detectable venom could persist for two weeks, which was associated with prolonged coagulopathy. Venom-induced consumption coagulopathy (VICC) is venom induced activation of the clotting pathway by procoagulant toxins, leading to consumption of clotting factors and coagulopathy. Venom-induced consumption coagulopathy is the most common systemic effect of snake envenomation; it varies from asymptomatic to fatal massive bleeding [9, 10]. Antivenom is still the most effective treatment to restore VICC and should be administrated as soon as possible. Unfortunately, antivenom has not always been available worldwide, especially in rural areas [11]. The effectiveness of antivenom on recovery of VICC is still controversial [12–14]. *Agkistrodon halys* antivenom (AHA, Shanghai Institute of Biological Products in China), a monospecific antivenom, is commonly used antivenom owing that there is no specific Green Pit Viper Antivenom (GPVA, The Thai Red Cross Society, Thailand) available in China. *Agkistrodon halys* antivenom is used to treat GPV envenomation owing to its in-vitro cross-neutralization effect on GPV venom. In-vivo mortality study showed that the neutralization capacity of AHA was super to GPVA [15]. But the improvement of coagulation dysfunction is unsatisfactory after AHA administration [16].

Protocols to prevent bleeding and improve recovery of VICC are essential in the treatment of Viperidae snakebites. The liver needs three to nine hours to re-synthesise clotting factors [17]. Clotting factor replacement has been used to improve coagulopathy. Fresh-frozen plasma (FFP) is the most commonly used clotting factor because it contains a variety of coagulation factors and it is easily available throughout the world [18]. Based on a few animal experiments [19], it is believed that clotting factor replacement would provide additional substrate for unneutralized venom, which may be dangerous in the early stage of snakebite. It is recommended to give FFP for actively bleeding patients. But some studies showed that routine clotting factor replacement might facilitate the recovery of the coagulation parameters in snakebite patients after antivenom administration. Brown et al. reported that early coagulation factor replacement after administering the antivenom was associated with early recovery of coagulopathy [20]. A randomized controlled trial by Isbister et al. showed that FFP transfusion after antivenom administration resulted in faster clotting function restoration in most patients. But there was no decrease in hospital stay [21]. Holla et al. suggested that plasma transfusion can help to restore coagulation functions rapidly and reduce the amount of antivenom [22].

Hence, clotting factor replacement is used as adjunct therapy to antivenom in hope to improve recovery of coagulopathy and reduce bleeding events in patients with VICC in China. However, given the lack of evidence on the effects of clotting factor replacement, this retrospective study aimed to investigate the effectiveness of clotting factor replacement for VICC in patients with GPV bites after antivenom administration.

## Methods

**Fig. 1:**
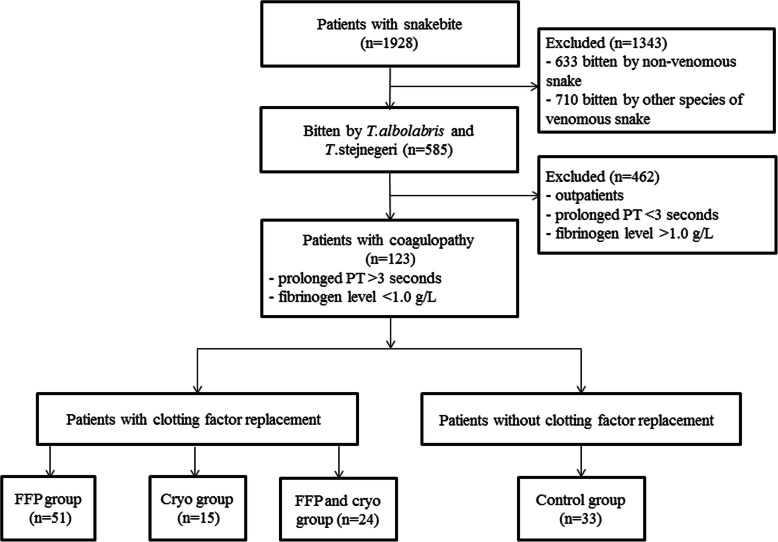
Study flowchart

In this study, we retrospectively reviewed patients (children under 14 years old and pregnant women were excluded) who were bitten by *T. albolabris* and *T. stejnegeri* and were admitted to our Emergency Department between 2013 and 2019. Our hospital is a tertiary teaching hospital of 1500 beds and is the major institution for treating venomous snakebite in Guangzhou, Southern China. The study protocol was approved by the ethics committees of the First Affiliated Hospital of Guangzhou Medical University (2018 No.K-23). Written informed consent to replacement therapy was obtained from participants of legal age and the parents or legal guardians of children (under16 years old). The study protocol is performed in accordance with the relevant guidelines. All objective clinical parameters were recorded using a pre-formatted clinical data form that included sociodemographic data, snake species, epidemiological data and various pertinent laboratory results such as complete blood count (CBC), blood urea nitrogen (BUN), creatinine, serum potassium, PT, fibrinogen, activated partial thromboplastin time (APTT) and D-dimer. Additionally, the type and dose of antivenom used, the type and dose of clotting factor used, as well as their adverse effects and outcomes were included in this form. Identification of venomous species was based on dead or live snake brought in or on-site photography of snakes presented to the hospital. The patients who only witnessed the offending snakes were asked to identify snake species using preserved specimen and color pictures. Patients bitten by other species of venomous snakes and non-venomous snakes were excluded. Among patients bitten by *T. albolabris* and *T. stejnegeri*, patients without coagulopathy and outpatients were also excluded. A total of 123 patients bitten by *T. albolabris* and *T. stejnegeri* with coagulopathy were included in the analysis (Fig. 1).

Previous studies indicated that prolonged PT was related to severe hypofibrinogenaemia in GPV bitten patients [23]. Fibrinogen level is useful to determine if the venom has been neutralised and the coagulopathy has resolved. The cut-off value of prolonged PT>3 s has been accepted to be indicative for activation of coagulation pathways [24]. And a fibrinogen level of 1.0 g/L has been accepted to be sufficient for haemostasis [18, 23, 24]. The normal reference range of PT was 11.0 ~ 14.5 s in our hospital. Patients with coagulopathy were defined as prolonged PT > 3 s to unmeasurable, and hypofibrinogenemia (fibrinogen level ranging from < 1.0 g/L to undetectable). Treatment for snakebites included antivenom, anti-tetanus, steroids, antibiotics and clotting factor replacement therapy. According to the package insert of AHA, it neutralizes venom of green pit vipers as well as *Agkistrodon halys*. The recommended initial dose was one vial (6000 IU) which is expected to neutralize the average venom yield. The same dose was repeated six hours after the initial dose of antivenom owing to persistent or recurrent coagulopathy [17]. For all patients with coagulopathy after antivenom administration, clotting factor (FFP or cryoprecipitate) transfusions were considered. But only the patients agreed and signed the informed consents were treated with clotting factor transfusions. The patients who signed consent to refuse clotting factor replacement were selected as control group, which was accorded with the principle of ethics. Patients treated with clotting factors were defined as clotting factor replacement therapy group, and were subdivided into three groups: (1) FFP group; (2) cryoprecipitate (cryo) group; and (3) FFP and cryo group. Coagulopathy patterns and effects of clotting factor replacement on clotting parameter were compared among four groups. Both patients in the control group and the other three clotting factor replacement groups satisfied the same inclusion criteria above.

Coagulopathy patterns were classified into the following categories: (1) stabilized, which was defined as the values corrected following the first antivenom administration within 48 h and did not recur; (2) persistent, which was defined as the values did not return to normal by the time of discharge; (3) recurrent, which was defined as the values were abnormal within the first 12 h, became normal, and then returned to abnormal; (4) late, which was defined as the values were abnormal since 12 h or more following the first antivenom administration and did not return to normal within 48 h [25].

Continuous data were presented as mean ± standard deviations (mean ± *SD*) or median (interquartile rang, IQR) (according to the Kolmogorov-Smirnov test) and between group comparison used the independent sample t test or Mann-Whitney U test. One-way analysis of variance was used to compare the four groups and Student Newman-Keuls was used to compare multiple groups. Categorical data were presented as frequencies or percentages and between groups comparison used the chi-square or Fisher exact test. The recovery of clotting parameters was calculated using the Kaplan-Meier method, in which the endpoint was defined as PT improved (prolonged PT < 3 s) and fibrinogen level improved (fibrinogen level > 1.0 g/L). The time was defined as the period from the initial dose of antivenom administration to 14 days. Comparison of percentage of unimproved coagulopathic parameters of the distinct groups was analyzed using the log-rank and Gehan-Breslow-Wilcoxon tests. The data were analyzed using SPSS 16.0 (IBM, Armonk, NY, USA) and Prism 7 (GraphPad, La Jolla, CA). A two-sided *P* value< 0.05 was considered statistically significant.

## Results

### Snake identification

Of the 1928 snakebite victims admitted during the study period, nine species of venomous snakes were identified as stated earlier. The species of venomous snakes included GPVs (*T. albolabris* and *T. stejnegeri*) (585 patients), *Naja atra* (290 patients), *Bungarus multicinctus* (65 patients), *Daboia siamensis* (54 patients), *Protobothrops mucrosquamatus* (19 patients)*, Deinagkistrodon acutus* (15 patients), *Ophiophagus hannah* (14 patients), *Rhabdophis subminiatus* (4 patients) and *Bungarus fasciatus* (1 patients). Coagulopathy (prolonged PT > 3 s and fibrinogen level < 1.0 g/L) was found in 31% (181/585) of patients following GPVs bite.

### Epidemiological features

Among 123 patients bitten by *T. albolabris* and *T. stejnegeri* with coagulopathy, the mean age of patients was 49 ± 16.6 years (range: 14–82 years); 61.0% (75/123) were male. Most bites (33.3%, 41/123) occurred in September. A total of 52.8% (65/123) of patients were admitted within six hours after snakebite. The sites of snakebite occurred in extremities in 99.2% (122/123) of patients, and the most common site of bite was found at the right extremity in 55.3% (68/123) of patients. Local swelling and pain was found in all patients. Most patients (87.0%, 107/123) had two fang punctures. Ecchymosis within the affected extremity was present in 96.7% (119/123) of patients, and ecchymosis extending beyond affected extremity was found in 3.3% (4/123) of patients. The length of hospital stay was 6 days (IQR 3 to 9 days). The baseline information of the patients was similar among the four groups (Table 1).

**Table 1:** Baseline characteristics of the patients with and without clotting factor replacement

Characteristics	FFP group	Cryo group	FFP and cryo group	Control group	*P* value
	(*n* = 51)	(*n* = 15)	(*n* = 24)	(*n* = 33)	
Age, year, (mean ± *SD*)	48 ± 16.2	52 ± 16.7	52 ± 15.7	48 ± 18.4	0.774
Male sex, n (%)	29/51 (56.9)	8/15 (53.3)	19/24 (79.2)	19/33 (57.6)	0.238
WBC, × 10^9^/L, (mean ± *SD*)	12.4 ± 4.4	13.4 ± 6.5	13.8 ± 6.6	12.0 ± 4.2	0.550
Hemoglobin, g/dL,(mean ± *SD*)	139 ± 16.8	134 ± 28.7	132 ± 29.0	135 ± 18.4	0.621
Platelets, ×109/L,(mean ± *SD*)	163.5 ± 75.1	156 ± 75.2	123 ± 62.3	167 ± 51.2	0.065
BUN, mmol/L, (mean ± *SD*)	5.3 ± 1.9	5.1 ± 1.5	5.9 ± 2.9	4.8 ± 1.9	0.305
Creatinine, μmol/L, (mean ± *SD*)	81.4 ± 22.8	70.4 ± 15.9	91.6 ± 37.1	79.3 ± 19.5	0.079
Serum potassium, mmol/L, (mean ± *SD*)	3.6 ± 0.3	3.6 ± 0.4	3.7 ± 0.3	3.7 ± 0.4	0.468
D-dimer,ng/mL, (mean ± *SD*)	858 ± 481.9	685 ± 381.0	837 ± 636.0	688 ± 520.0	0.486
Time of antivenom post-bite	24/51 (47.1)	10/15 (66.7)	9/24 (37.5)	22/33 (66.7)	0.085
(≤6 h), n (%)					
Amount of antivenom,vial,(mean ± *SD*)	2 ± 0.9	2 ± 1.2	2 ± 0.5	2 ± 0.8	0.348
Hours of clotting factor given after admission,hour,(*mean* ± *SD*)	41 ± 38.2	36 ± 35.6	24 ± 34.0	–	0.269

*FFP* fresh-frozen plasma, *cryo* cryoprecipitate, *SD* standard deviations, *WBC* white blood cells, *BUN* blood urea nitrogen

### Clinical profile

Of the 123 patients, 90.2% (111/123) received AHA after admission to our hospital, and 9.8% (12/123) had received AHA at a local hospital before admission. The median amount of antivenom used was 2 vials (IQR 2 to 2 vials). Local symptoms of envenomation such as swelling and pain were improved rapidly after antivenom infusion.

**Fig. 2:**
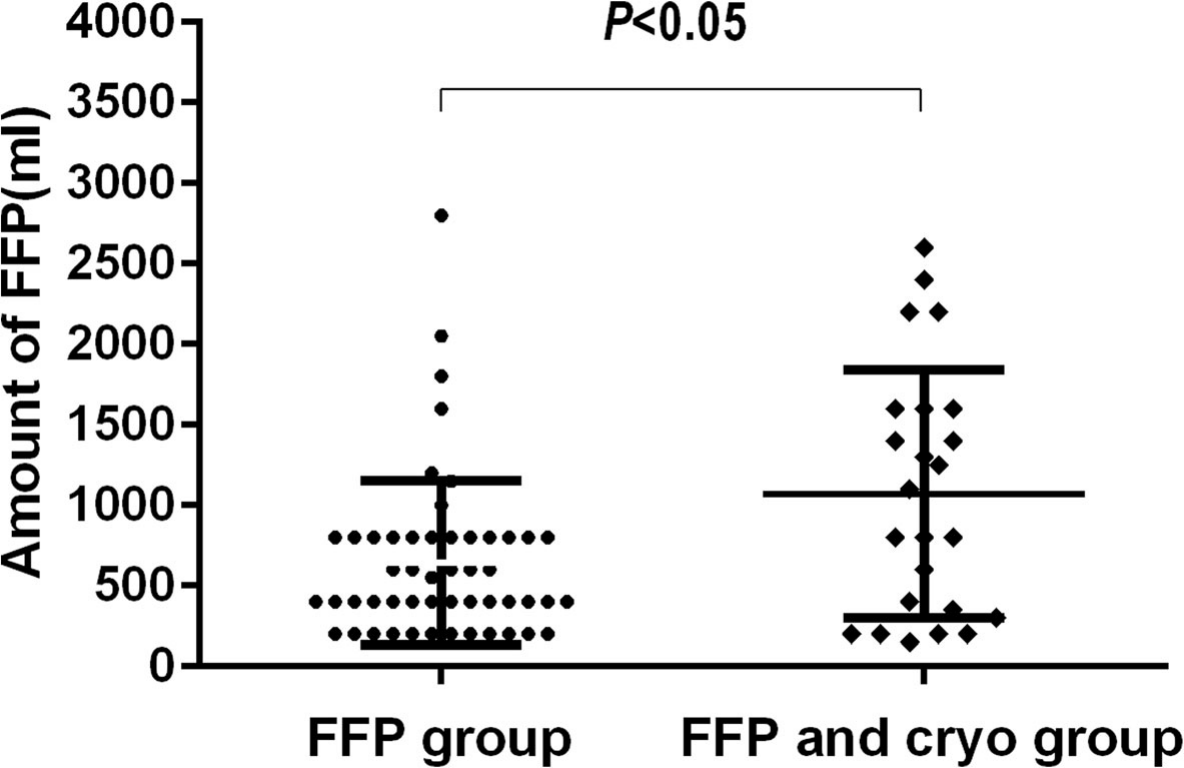
The amount of FFP transfused in the FFP group and the FFP and cryo group. There was significant difference between the amounts of FFP transfusion in the two groups (*P* < 0.05)

Among 90 patients who received clotting factor replacement, there were 51 patients in the FFP group, 15 patients in the cryo group, and 24 patients in the FFP and cryo group, respectively. The median time of the initial dose of clotting factor transfusion after admission was 21 h (IQR 7 to 52 h). The median amount of FFP transfusion was 400 ml (IQR400 to 800 ml) in the FFP group, and 950 ml (IQR310 to 1600 ml) in the FFP and cryo group, respectively. There was significant difference between the amounts of FFP transfusion in the two groups (*P* < 0.05) (Fig. 2). The median amount of cryo transfusion was 10 ± 7.2 units in the cryo group, and 8 ± 6.7 units in the FFP and cryo group, respectively.

**Fig. 3:**
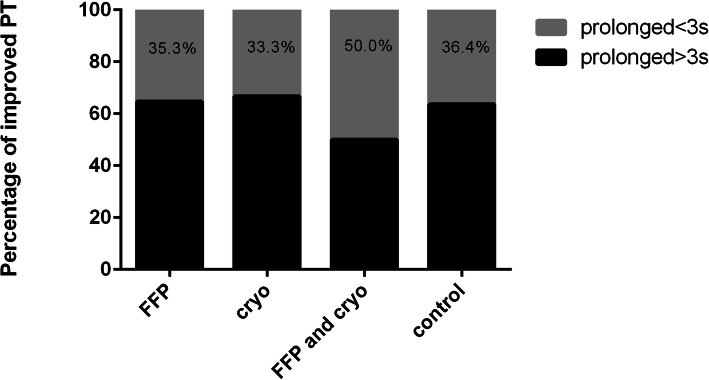
Percentage of improved PT 14 days after antivenom administration. There were no significant differences among the four groups (*P* > 0.05)

**Fig. 4:**
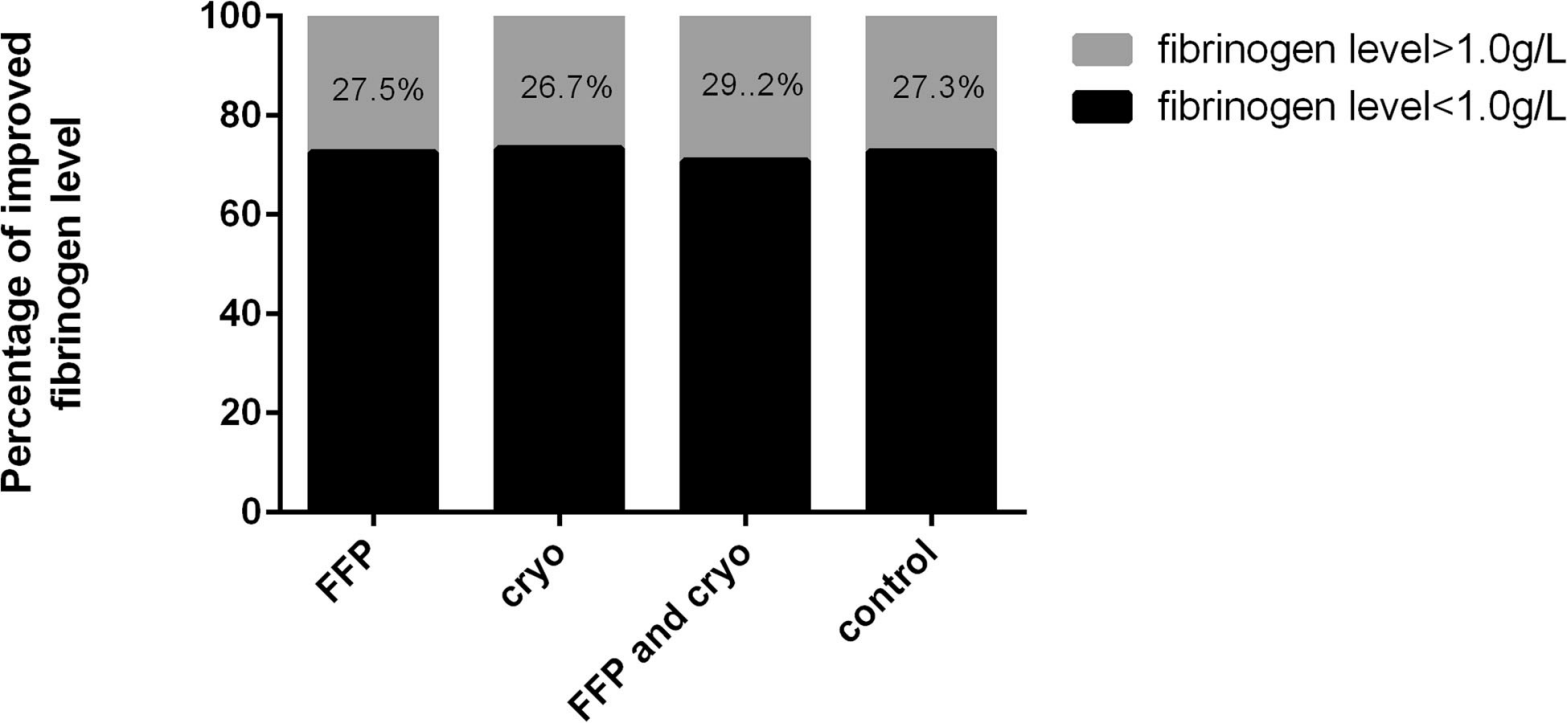
Percentage of improved fibrinogen level 14 days after antivenom administration. There were no significant differences among the four groups (*P* > 0.05)

Coagulopathy patterns of patients with and without clotting factor replacement after antivenom administration showed that persistent and late coagulopathy were the most common patterns among four groups. No statistical significance of persistent and late coagulopathy was found among four groups (PT: *P* = 0.21; fibrinogen level: *P* = 0.42) (Table 2). The effect of clotting factor transfusions on PT and fibrinogen level was shown in Table 3. The median reduction in PT was 20.1 ± 31.2 s for the FFP and cryo group. There was significant difference between the cryo group and the FFP and cryo group (*P* = 0.02). The median increase in fibrinogen level was very small: 0.05 ± 0.20 g/L for the FFP group, 0.09 ± 0.37 g/L for the cryo group and 0.07 ± 0.31 g/L for the FFP and cryo group, respectively. No significant differences in improved PT (prolonged PT < 3 s) were observed among the four groups 14 days after initial antivenom (Fig. 3). Similarly, no significant differences in improved fibrinogen level (fibrinogen level > 1.0 g/L) were found among the four groups (Fig. 4).

**Table 2:** Coagulopathy patterns of the patients with and without clotting factor replacement treatment

Characteristics		FFP group		Cryo group		FFP and cryo group		Control group
		(*n* = 51)		(*n* = 15)		(*n* = 24)		(*n* = 33)
	PT	Fibrinogen	PT	Fibrinogen	PT	Fibrinogen	PT	Fibrinogen
	n(%)	n(%)	n(%)	n(%)	n(%)	n(%)	n(%)	n(%)
Stabilized	4 (8)	3 (6)	2 (13)	0	1 (4)	0	2 (6)	0
Persistent	20 (39)	27 (53)	6 (40)	12 (80)	11 (46)	15 (63)	8 (24)	20 (61)
Recurrent	2 (4)	1 (2)	0	0	4 (17)	2 (8)	2 (6)	0
Late	25 (49)	20 (39)	7 (47)	3 (20)	8 (33)	7 (29)	21 (64)	13 (39)

Coagulopathy patterns were classified as follow:

(1) stabilized: the values corrected following the first antivenom administration within 48 h and did not recur;

(2) persistent: the values did not return to normal by the time of discharge;

(3) recurrent: the values were abnormal within the first 12 h, became normal,and then returned to abnormal;

(4) late: the values were abnormal since 12 h or more following the first antivenom administration

Rows of stabilized and recurrent were excluded for analysis due to 50% cells have expected count less than 5

No statistical significance of persistent and late coagulopathy were found among four groups(*P* = 0.21 and *P* = 0.42, respectively)

*FFP* fresh-frozen plasma, *cryo* cryoprecipitate, *PT* prothrombin time

**Table 3:** Changes in values for PT and fibrinogen level after transfusion of FFP and cryo

Characteristics	PT(s)		Fibrinogen level(g/L)	
	Reduction		Increase	
	n	mean ± *SD*	n	mean ± *SD*
FFP group	43^a^	6.3 ± 15.2	51	0.05 ± 0.20
Cryo group	13^b^	0.4 ± 18.9	15	0.09 ± 0.37
FFP and cryo group	23^c^	20.1 ± 31.2^*^	24	0.07 ± 0.31

^abc^A totle of 11 patients were excluded from analysis due to unmeasureable posttransfusion/pretransfusion PT.

*There was significant difference between the FFP and cryo group and the cryo group(*P* = 0.02)

*PT* prothrombin time, *FFP* fresh-frozen plasma, *cryo* cryoprecipitate, *SD* standard deviations

The length of hospital stay was 6 ± 3.5 days in the FFP group, 6 ± 3.2 days in the cryo group, 9 ± 4.4 days in the FFP and cryo group, and 5 ± 3.0 days in the control group, respectively. The FFP and cryo group had a significantly longer length of hospital stay than the other groups (*P* < 0.05).

**Fig. 5:**
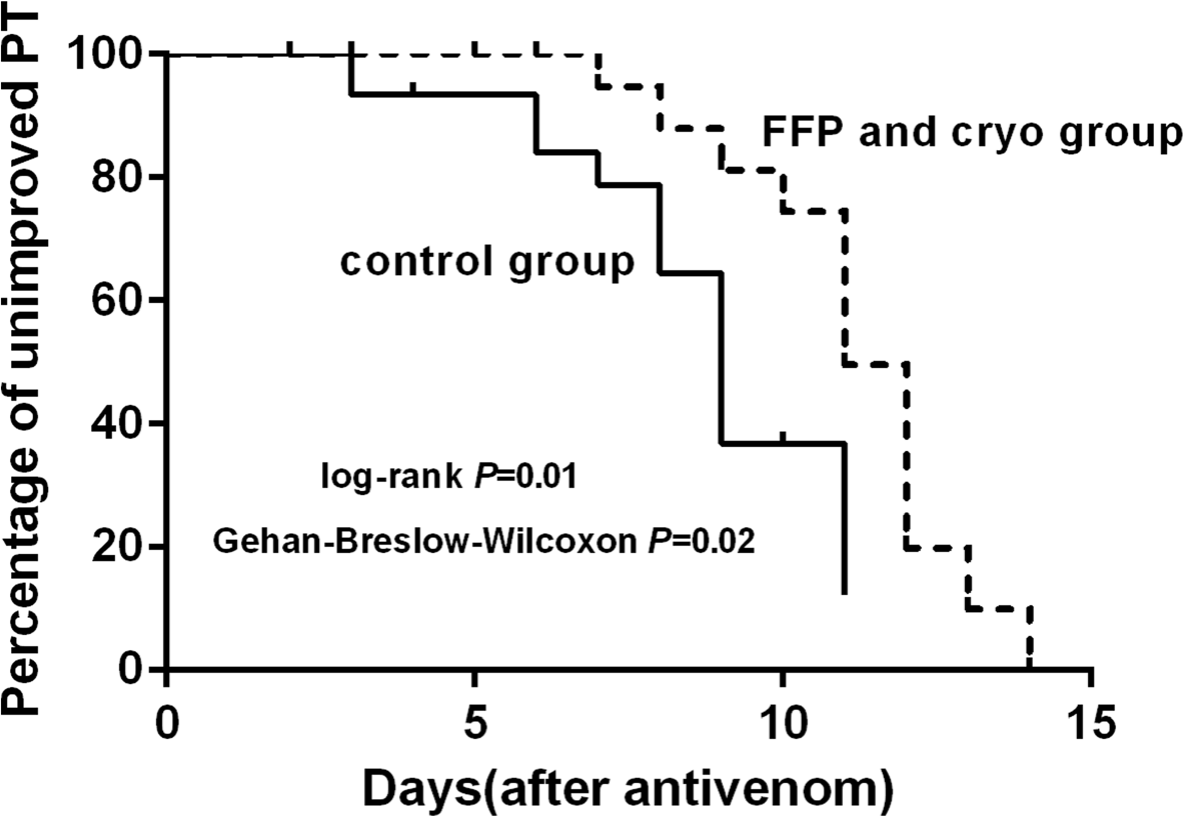
A survival curve of time to unimproved PT (prolonged PT > 3 s) comparing with patients received FFP and cryo to those without. Kaplan-Meier analysis indicated that the percentage of unimproved PT was markedly higher in the FFP and cryo group than that in the control group (*P* = 0.01 by log-rank test, *P* = 0.02 by Gehan-Breslow-Wilcoxon test)

Survival analysis showed that no significant difference of unimproved PT between the FFP group and the control group (*P* = 0.23 by log-rank test, *P* = 0.92 by Gehan-Breslow-Wilcoxon test), and the cryo group and the control group (*P* = 0.67 by log-rank test, *P* = 0.64 by Gehan-Breslow-Wilcoxon test). The percentage of unimproved PT was markedly higher in the FFP and cryo group than that in the control group (*P* = 0.01 by log-rank test, *P* = 0.02 by Gehan-Breslow-Wilcoxon test) (Fig. 5). The median for survival time was 11 days (95% CI: 10–12) in the FFP and cryo group, and 9 days (95% CI: 8–10) in the control group.

**Fig. 6:**
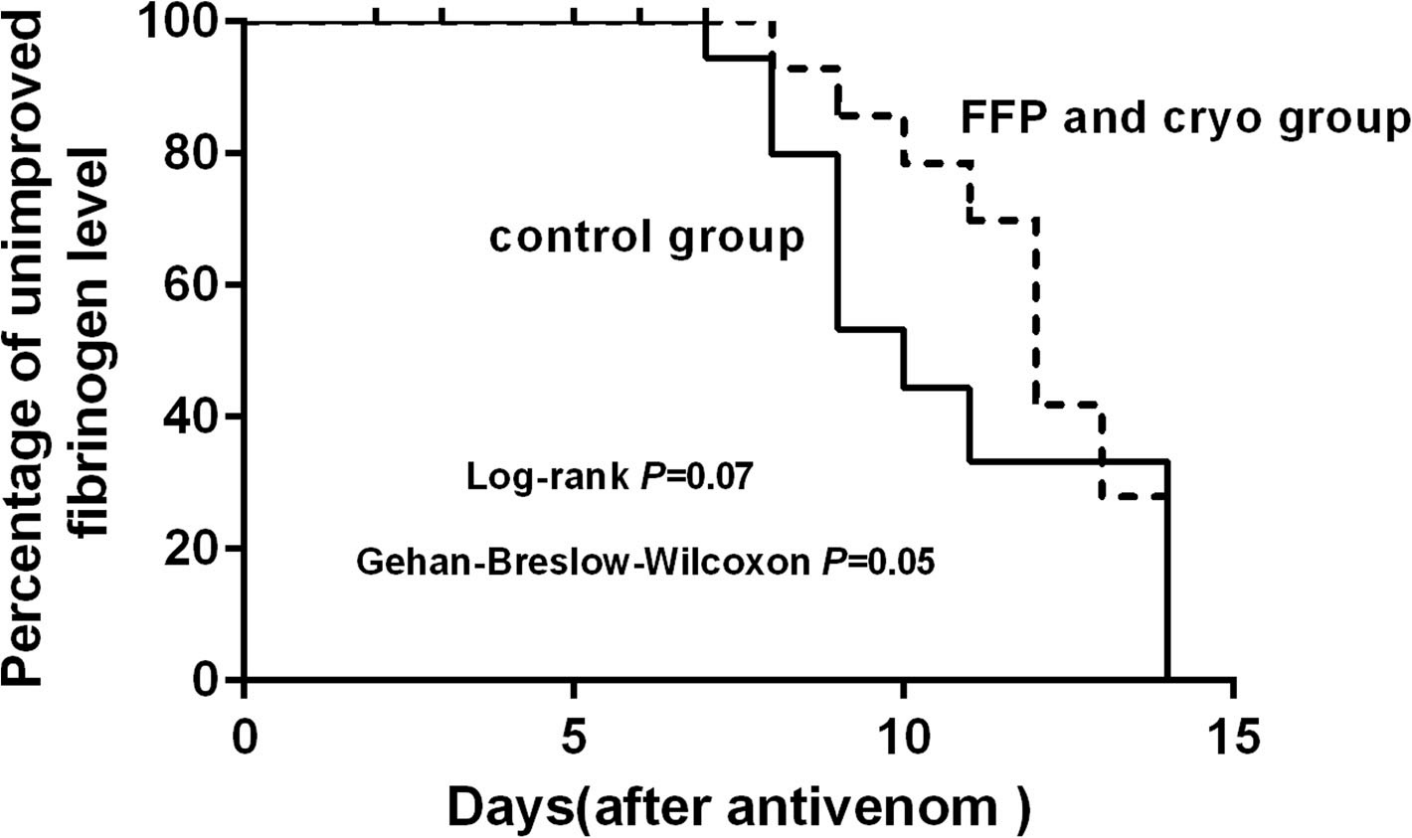
A survival curve of time to unimproved fibrinogen level (fibrinogen level < 1.0 g/L) comparing with patients received FFP and cryo to those without. Kaplan-Meier analysis indicated that the percentage of unimproved fibrinogen level tended to be worse in the FFP and cryo group than the control group, but the different was marginal (*P* = 0.05 by Gehan-Breslow-Wilcoxon test, *P* = 0.07 by log-rank test)

Survival analysis showed that no significant difference of unimproved fibrinogen level between the FFP group and the control group (*P* = 0.49 by log-rank test, *P* = 0.38 by Gehan-Breslow-Wilcoxon test), and the cryo group and the control group (*P* = 0.98 by log-rank test, *P* = 0.81 by Gehan-Breslow-Wilcoxon test). The percentage of unimproved fibrinogen level tended to be worse in the FFP and cryo group than the control group, but the difference was marginal (*P* = 0.05 by Gehan-Breslow-Wilcoxon test, *P* = 0.07 by log-rank test) (Fig. 6).

Enlargement or appearance of new ecchymosis were found in 5.6% (5/90) of the patients in clotting factor replacement groups and 6.1% (2/33) of the patients in the control group, respectively. No statistical significance was observed between two groups (*P* > 0.05).

One patient in the FFP and cryo group and another in the cryo group had an internal hemorrhage at admission. All patients developed VICC at admission and developed hemorrhage on day 6 post-bite by a GPV. One patient developed hepatic rupture and massive intra-abdominal hemorrhage. This patient was treated with selective right hepatic artery embolization and fully recovered. Another patient was diagnosed with massive hemorrhagic pleural effusion. The patient was treated with thoracic drain and also recovered completely. No internal bleeding was observed in the control group.

Five patients developed anaphylaxis with rash, itching, and fever after FFP transfusion, and another two elderly patients developed heart failure induced by FFP transfusion. Two patients from the control group were forced to stop administration of antivenom due to urticaria.

A total of ten patients in clotting factor replacement groups and three patients in the control group were lost to follow-up. All followed patients reported that local swelling improved or resolved without development of skin ecchymosis, gingival bleeding, and other manifestations of visceral hemorrhage.

## Discussion

Antivenom does not completely restore coagulopathy in all snakebites from venomous species. Antivenom can only act on the unbound free venom in circulation, while the combined venom has already caused a cascade of coagulation factor consumption. Many physicians sought alternative methods to bridge the lack of antivenom effectiveness on coagulopathy status post snakebites. Clotting factor replacement after antivenom therapy has been used to address coagulopathy however its effectiveness is still under investigation [20–22, 26]. In this present study, we retrospectively reviewed 123 patients with coagulopathy after GPV bites. Our study showed that administration of clotting factors after antivenom injections has no effect on the improvement of VICC in patients with GPV bites. Although GPV bite rarely causes death, it induces coagulation dysfunction [7, 27], which leads to increased risk of bleeding. Different from previous report [2], severe coagulation disturbances were common in our study. The absence of bleeding does not indicate the absence of VICC. The indicators for evaluating the efficacy of therapy are mainly based on the degree and speed of recovery of coagulation parameters as well as any bleeding incidents, rather than on the reduction of morbidity and mortality.

The present study showed that administration of clotting factors did not result in rapid restoration of clotting parameters and shorter hospital stay, which are not consistent with previous studies [20–22]. In the study of Isbister et al., FFP given after antivenom resulted in more rapid restoration of clotting function in most patients. The average time between antivenom administration and discharge ranged from 34 to 39 h [18]. Owing to the short observation period, transient or permanent improvement of parameters was unclear. It is unknown whether coagulation parameters worsened again after 48 h, since recurrence of coagulopathy is common in some venomous snakebite. Furthermore, the response to clotting factor replacement might be different among different species. In the present study, all patients were bitten by snakes of the same genus. It’s difficult for patients and clinicians to identify *T.albolabris* or *T.stejnegeri* by morphological characteristics. A small sample study in China showed that *T. albolabris* snakebites caused more severe coagulopathy and early onset of systemic bleeding than *T. stejnegeri* [28]. Over a longer observation period, we found that multiple clotting factor replacement did not improve coagulopathy comparing to those without clotting factor replacement. Our results were consistent with the study by Mion et al. which reported that administration of fresh frozen plasma and fibrinogen was inefficient in recovery of coagulation parameters in Viperidae bites [29]*.*

The infusion of the initial dose of clotting factor of the present study was 21 h after admission, which is significantly delayed comparing with the previous study [20, 21]. The reasonable time for infusion is controversial. Based on limited animal experiments [19], clotting factor is not recommended in the early stage of bite. Nevertheless, the present study suggests that delayed transfusion of clotting factors did not accelerate the improvement of abnormal coagulation parameters. All of these patients had received AHA based on evidence of cross neutralization of AHA and GPV venom. However, the lack of complete neutralization of GPV venom in circulation after AHA led to the presence of free venom in circulation. Hence, administration of FFP/cryoprecipitate would be undergoing consumption coagulopthy because free venom consumed added clotting factors and worsened the coagulopathy.

The optimal dose of FFP for patients with abnormal clotting test is unknown. The dose of FFP patients received in previous research was 10-15 mL/kg up to 1000 ml. In our study, the amount of FFP infusion in the FFP and cryo group was more than that in the FFP group. But more FFP seemed to make recovery of PT worse. More FFP had no added benefit over fewer FFP. We speculate that venom in circulation in the FFP and cryo group might consume more clotting factors and prolonged the recovery of coagulation parameters.

Clotting factor replacement is not a substitute for antivenom for treatment of VICC. Ideally, antivenom alone effectively reverses VICC, but antivenom can be insufficient in some cases. A study showed that neither the dose nor the time of antivenom reduced the time of recovery from coagulopathy [12]. In the present study, persistent and late coagulopathy were common in both clotting factor replacement groups and control group. It was important that VICC could not be reversed by antivenom binding to venom after the damage had occurred. Our study implied that the median two vials of AHA could not neutralize the GPV venom sufficiently. This may be partly attributed to the specificity of AHA. The specific GPV antivenom was superior to neutralization [16], but its availability is limited in China. AHA is a type of monospecific antivenom. Whether the large initial dose of AHA is beneficial in the management of patients following GPV bites is still unknown. In addition, the dose of antivenom is still controversial [30, 31]. The risk of reactions of high dose antivenom should always be taken into consideration.

In our study, hypofibrinogenemia was persistent in all groups. In order to increase the fibrinogen level, patients in the cryo group and the FFP and cryo group were repeatedly transfused with cryoprecipitate, which contained more fibrinogen than FFP, but its effect was not obvious. The median increase in posttransfusion fibrinogen level was generally very small and was almost negligible. Fibrinogen infusion might be more effective in improving hypofibrinogenemia. However, due to its higher price and low availability, the effect and significance of bleeding prophylaxis using fibrinogen in patients with GPV bites need further evaluation.

FFP transfusion is used widely to treat hematologic disorders. But it had no prophylactic effect on improving abnormal coagulation parameters in patients without bleeding or prior to an invasive procedure and operation [32]. Adverse reactions of clotting factor administration also cannot be ignored. Transfusion-associated circulatory overload (TACO) is the most frequent cause of death and major morbidity. In our study, we found that some patients suffered from the allergic reaction and TACO after FFP transfusion, and the latter may be due to aging and the amount of plasma transfused. Physicians need to weigh the risks and benefits of clotting factor replacement when managing snakebites.

### Limitations

The findings in this study do not support the use of clotting factors after antivenom administration in the management of coagulopathy. However, this study was a retrospective chart review, so the selection bias is inevitable. The small sample size is another limitation. Receiving both FFP and cryo could confound the effect of a single clotting factor therapy. The efficacy of a single factor needs to be further evaluated. There were wide variations in the doses of FFP and cryoprecipitate, which may further confound the evaluation of their effect. Since the severity of coagulopathy may vary among snake species, the results from GPVs may not be generalized to other species of venomous snakes.

## Conclusion

In conclusion, the clotting factor replacement therapy seems to have no effect on the improvement of coagulopathy in patients with GPV bites. Although GPV envenomation can lead to severe internal hemorrhage, the clinical benefit of prophylactic clotting factor replacement in those non-bleeding patients with abnormal clotting test was not obvious. The results from GPVs may not be generalized to other species of venomous snakes.

## Data Availability

Data are available from the corresponding author on reasonable request.

## Abbreviations

AHA: *Agkistrodon halys* antivenom
APTT: activated partial thromboplastin time
BUN: blood urea nitrogen
CBC: complete blood count
cryo: cryoprecipitate
FFP: fresh-frozen plasma
GPV: green pit viper
IQR: interquartile ranges
PT: prothrombin time
*SD*: standard deviations
TACO: transfusion-associated circulatory overload
*T. albolabris*: *Trimeresurus albolabris*
*T. stejnegeri*: *Trimeresurus stejnegeri*
VICC: venom induced consumption coagulopathy
WBC: white blood cells
